# Transcriptome Analysis of Differentially Expressed Genes in Freshwater Pearl Mussel (*Sinohyriopsis cumingii*) with Four Different Shell Colors

**DOI:** 10.3390/ani16030416

**Published:** 2026-01-29

**Authors:** Fuyong Huang, Qinghua Jiang, Jubin Xing, Yongbin Xu, Qingman Yang, Jinyu Tang, Zengping Tang, Xiao Liang, Shaohua Zhu, Bao Lou

**Affiliations:** 1 Institute of Hydrobiology, Zhejiang Academy of Agricultural Sciences, Hangzhou 310021, China; jqh881130@163.com (Q.J.);; 2 Zhuji Agricultural Technology Extension Center, Zhuji 311800, China; 3 Jinhua Fisheries Technology Extension Center, Jinhua 321000, China; 4 Shaoxing Fisheries Technology Extension Center, Shaoxing 312000, China; 5 Fishery Service Station of Wuyi County, Jinhua 321000, China; 6 Shaohua Pearl Farm, Wuyi County, Jinhua 321000, China

**Keywords:** *Sinohyriopsis cumingii*, color difference, transcriptomic analysis, pigment metabolism, mineral metabolism

## Abstract

Pearl culture has long been a major global industry. As a significant global producer, China mainly produces pearls from the freshwater mussel (*Sinohyriopsis cumingii*). *S. cumingii* with various shell colors can produce pearls of different colors; for example, mussels with a blue-white shell can produce white pearls, while those with a purple shell can produce light-purple pearls. Therefore, investigating the molecular genetics of shell color variation in *S. cumingii* can advance our understanding of the mechanisms underlying differences in shell and pearl coloration in these mussels. In this study, we selected juvenile *S. cumingii* with four differently colored inner shells and collected tissue samples for transcriptomic analysis. The results showed that many key genes involved in the regulation of pigment metabolism (such as ADAMTS, TYR, BCDO2, and FTH1), as well as those associated with metabolism and mineral absorption (such as TRPV6, HCP1, HEPH, and Zip4), exhibited significant differences. Furthermore, these DEGs (differentially expressed genes) may influence the synthesis and metabolism of melanin, carotenoids, porphyrins, and heme, thereby affecting shell color variation; they might also be one of the potential reasons why *S. cumingii* produces pearls of different colors.

## Simple Summary

Pearl culture is a significant global industry, with China being a leading producer in utilizing the freshwater mussel *Sinohyriopsis cumingii*. The color of the pearl is closely linked to the shell color of the mussel, yet the molecular genetic basis driving this variation remains unclear. This study investigated the mechanisms underlying shell and pearl coloration by analyzing the transcriptomes of juvenile *S. cumingii* with four distinct inner-shell colors. The results revealed significant differential expression of key genes regulating pigment metabolism, and genes involved in mineral metabolism and absorption. These findings indicate that shell color phenotypes are under stable genetic control, with the identified genes directly influencing color formation through pigment deposition and mineral integration. This study shows that specific genetic pathways govern color variation in *S. cumingii*, providing a molecular foundation for selective breeding. This knowledge holds considerable social and economic value, as it enables the targeted cultivation of mussels to produce pearls with desirable and consistent colors, thereby enhancing product quality, market value, and sustainability for the global pearl industry.

## 1. Introduction

Pearl cultivation is a major global aquaculture industry, with China leading in freshwater pearl production. In 2019, China accounted for approximately 90% of the world’s output, primarily through the farming of *Sinohyriopsis cumingii*, a freshwater mussel native to China [1,2]. This species is highly productive and yields high-quality pearls in various colors, including white, pink, yellow, and purple, with the latter being especially popular in the market [3,4]. Its ability to produce diverse pearl colors is likely due to extensive artificial breeding, which has enriched its genetic diversity. Studies suggest that selecting donor mussels based on their inner-shell color can help cultivate pearls of corresponding colors [5]. Therefore, investigating the genetic link between shell color and pearl color in *S. cumingii* has become a key research focus in the industry.

Numerous studies have shown that pearls and shells are very similar in structure and composition; both are influenced by biomineralization during secretion and formation [6,7,8]. Pearls form when foreign substances are encapsulated by the pearl layer substance of a shell; over a long period, adhesion, consolidation, and proliferation occur, and a complete pearl is eventually created, similar to the formation process of otoliths in Sciaenidae [9]. The main component of the pearl layer is calcium carbonate, along with some organic matter, which is the same as that of the shell; this small amount of organic matter often determines the color of the pearl, similar to the color formation process of shells. However, research on the color differences in the inner shells has mainly focused on the synthesis, metabolism, and deposition processes of various pigment components [3,4,5,6]. Numerous studies have shown that the levels of melanin and carotenoids in shellfish directly or indirectly affect shell color [10,11,12,13,14], and this is thus regarded as a key factor influencing pearl color. Therefore, if a single shell color of *S. cumingii* could be precisely selected and bred, it would be possible to achieve targeted cultivation of pearls with a single color, thereby significantly enhancing the economic benefits of pearl culture. In recent studies, the genetic traits of *S. cumingii* have been analyzed through genomic research and genome-wide association studies (GWASs). Some researchers have used deep sequencing to assemble and analyze the chromosomal-level genome of *S. cumingii*, gaining new insights into the biological mineralization mechanisms of pearls [3]. Other researchers have used GWAS to deeply analyze genetic molecular markers of purple *S. cumingii* to selectively breed purple-shelled *S. cumingii* [4]. These studies have provided many new insights into the genetics and breeding of *S. cumingii*.

Based on the aforementioned studies, we identified another research topic. In traditional breeding areas in China, differently colored shells of the triangle sail mussel can be found simultaneously in the same fishpond. During the breeding process, significant variation in the shell colors of *S. cumingii* can be observed with the human eye, even at the larval stage. This phenomenon indicates that under traditional breeding models (models without artificial selection), natural reproduction in *S. cumingii* shows high morphological diversity. These differences in shell color have become stable phenotypic traits, suggesting that after a long and extensive breeding process, *S. cumingii* may have evolved into multiple stable differential varieties. Therefore, we speculate that fully understanding and analyzing the genetic information and distinctive characteristics of differently colored inner shells of *S. cumingii* ewould help improve the efficiency of targeted breeding for species with distinctive pearls and increase their industrial value.

In this study, we selected juvenile *S. cumingii* with four differently colored inner shells and collected tissue samples for transcriptomic analysis. Furthermore, we conducted a correlation analysis based on the published research results of our peers to enrich the genetic background information on shell color differences. This is expected to contribute to the development of a specialty pearl-oriented breeding industry.

## 2. Materials and Methods

### 2.1. Animals

**Figure 1:**
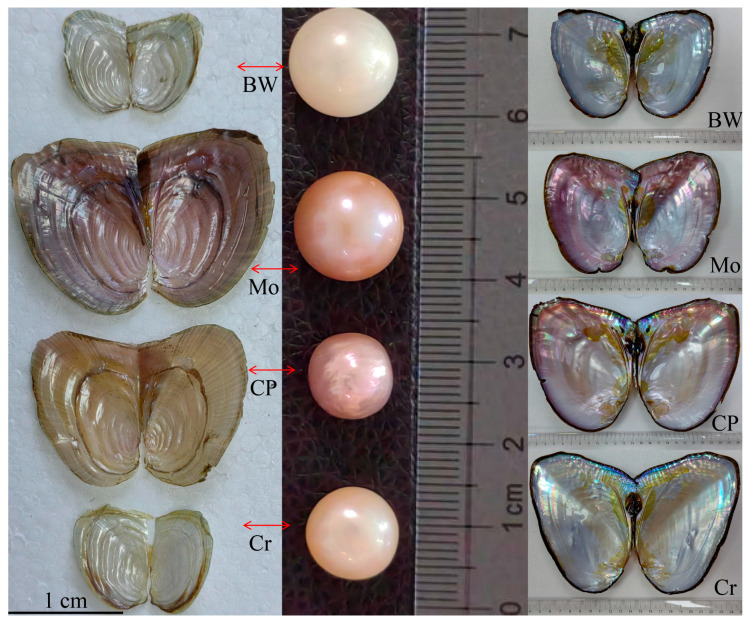
Four different shell colors and pearl colors in *Sinohyriopsis cumingii*. The colors were blue-white (BW), modena (Mo), seven-color purple (CP), and crystal transparency (Cr). The left side of the figure displays four species of juvenile of *S. cumingii* collected for this study, while the right side of the figure displays four types of adult *S. cumingii* with distinct color variations and their corresponding pearls.

We collected samples of four different inner-shell color varieties of juvenile *S. cumingii* in June, from the Shaohua Pearl Farm in Wuyi County, Jinhua City, Zhejiang Province, China. The parental *S. cumingii* used were classified according to color differences in their shells and pearls before the breeding process began. These juveniles were first placed on the gills or tail fins of yellow catfish (*Pelteobagrus fulvidraco*) in greenhouse facilities to facilitate parasitism and promote metamorphosis, transforming the *Glochidium* stage into normal juveniles. Once they reached a stage at which they could freely detach, they were then transferred to an external pond for further breeding. The shell diameter of the collected larvae ranged from 0.65 cm to 1.2 cm, and their weight ranged from 0.8 g to 1.3 g. A total of 12 samples (every color variety had three samples) were used for RNA sequencing (RNA-seq). Four differently colored inner-shell *S. cumingii* had obvious color differences, and we named them according to the colors of their appearance and shells, including blue-white (BW), modena (Mo), seven-color purple (CP), and crystal transparency (Cr) (Figure 1: the left side of the figure displays four types of juvenile *S. cumingii* that had been collected). All tissues from every juvenile *S. cumingii* were collected, weighed (range = 0.5–1.0 g each sample), and stored at −80 °C until RNA extraction.

### 2.2. Extraction of Total RNA

Total RNA was extracted and isolated from all samples using QIAzol Lysis Reagent (Qiagen, Dusseldorf, Germany). The quantity and purity of total RNA were confirmed with a NanoDrop 2000 Spectrophotometer (Thermo Fisher Scientific, Altrincham, Cheshire, UK). We used agarose gel electrophoresis to evaluate RNA integrity, and RIN numbers were measured by using an Agilent 5300 (Agilent, Santa Clara, CA, USA). In the end, all RNA samples were stored at −80 °C until use.

### 2.3. RNA Sequencing

For RNA-seq, total RNA was first enriched for poly(A) RNA using Oligo(dT) magnetic beads. Subsequently, RNA-seq libraries were prepared from approximately 1 µg of enriched RNA using the Illumina^®^ Stranded mRNA Prep Kit (Illumina, San Diego, CA, USA). Sequencing was completed using the Illumina Novaseq 6000 platform by Meiji Biotechnology Co., Ltd. (Majorbio, Shanghai, China) (https://www.majorbio.com/) (accessed on 26 August 2025).

### 2.4. Alignment of Transcriptomic Data

All raw sequences were analyzed by base mass, base error rate, and base content to remove low-quality sequences. Then, high-quality sequences were mapped to the *S. cumingii* reference genome [3] using TopHat2 v2.1.1 and HISAT2 v2.0.5 [15,16]. Then, the mapped reads assembling and function annotation were completed using Cufflinks v0.8.0 software [17]. The total number of reads for each gene was generated by the RSEM v1.3.3 software [18]. DESeq2 v1.42.0 software was used for the analysis of differential gene expression [19]. Genes with a false discovery rate (FDR) value ≤ 0.05 and an absolute value of log2 (fold change) ≥ 1 were defined as DEGs.

### 2.5. GO and KEGG Pathway Enrichment Analysis

Protein sequence of the obtained genes were subjected to BLAST searches against SwissProt using DIAMOND v2.1.8 (https://github.com/bbuchfink/diamond, accessed on 26 August 2025) [20], followed by analyses of COG (Clusters of Orthologous Groups of Proteins), GO (Gene Ontology), and KEGGs (Kyoto Encyclopedia of Genes and Genomes). Meanwhile, Goatools v0.7.6 (https://github.com/tanghaibao/GOatools, accessed on 26 August 2025) [21] was used to finish GO term enrichment analysis, and KEGG pathway enrichment analysis was completed by using the R package clusterProfiler v4.0 [22].

### 2.6. Expression Analysis of DEGs via qRT-PCR

After total RNA extraction, some samples were used for first-strand cDNA synthesis. Frst, genomic DNA was removed using 10 × gDNA Remove Mix (Takara, Kyoto, Japan). Then, cDNA synthesis was completed according to the manufacturer’s protocol of the Hifair^®^ II 1st Strand cDNA Synthesis Kit (Yeasen, Shanghai, China). Next, quantitative real-time PCR (qRT-PCR) was performed on an ABI 7500 qPCR instrument (Thermofisher, Sunnyvale, CA, USA) using the SYBR^®^ Premix Ex Taq™ II Kit (Takara, Kyoto, Japan) according to the manufacturer’s instructions. The PCR program was set as follows: 95 °C for 2 min, followed by 40 cycles of 95 °C for 15 s and 56 °C for 20 s. All reactions were carried out in triplicate as technical replicates, with *S. cumingii* β-actin used as the reference gene. Relative mRNA expression levels were calculated using the comparative Ct (2^−∆∆Ct^) method [23]. Primers were designed using Primer v5.0 software. Differential characteristics were analyzed using one-way analysis of variance (ANOVA) with SPSS v24.0 software (IBM, Armonk, NY, USA).

## 3. Results

### 3.1. Analysis of Transcriptomic Data

In this study, twelve transcriptome libraries were obtained, generating 73.47 Gb of clean data. Each library totaled more than 5.75 Gb of clean data, and Q30 values were over 96.69% (Table 1). After evaluating the assembly results, 96,804 unigenes and 136,658 transcripts were obtained, with an average N50 length of 1985 bp. All these genes were matched against the Kyoto Encyclopedia of Genes and Genomes (KEGGs), the Pfam database, the Non-Redundant Protein Sequence Database (NR), the Evolutionary Genealogy of Genes: Non-supervised Orthologous Groups (eggNOG), the Swiss-Prot database, and the Gene Ontology (GO) database. At the end, a total of 15,733 genes were identified in these databases, including 9265 known DEGs (Tables S1 and S2). All raw sequence data were deposited in the National Center for Biotechnology Information (NCBI) Sequence Read Archive (SRA) (see the “Data Availability Statement” for details).

### 3.2. Analysis of Differentially Expressed Genes

**Figure 2:**
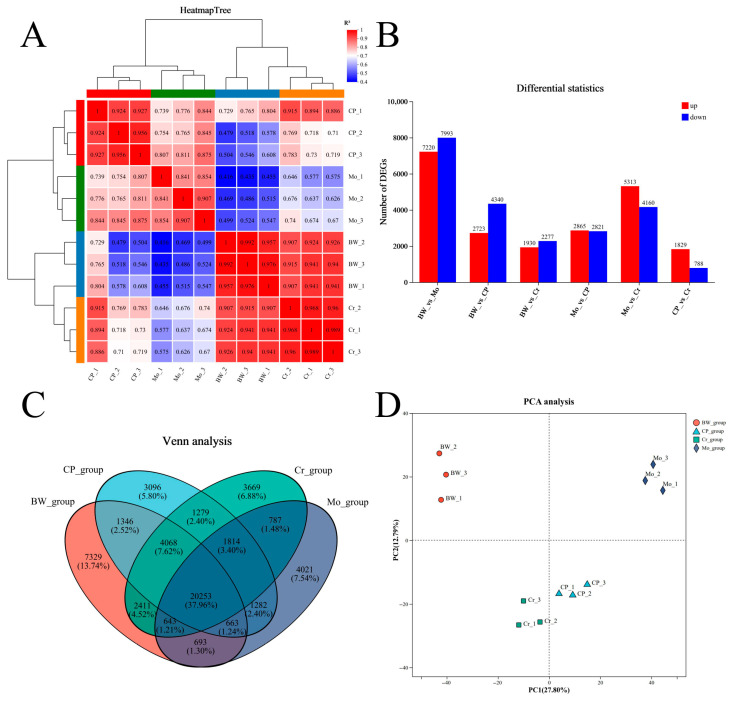
Transcriptomic data analysis. (**A**) Correlation analysis between biological replicate samples; (**B**) differential gene comparisons among six comparison groups; (**C**) Venn analysis of four groups; (**D**) principal component analysis (PCA).

Through sequencing analysis, we finally obtained six comparison groups. The correlation analysis is shown in Figure 2A. The differential statistics of the DEGs are shown in Figure 2B, and the results are shown in the form of Venn diagram in Figure 2C, and the results of the principal component analysis (PCA) are shown in Figure 2D. The group with the most DEGs was the BW_vs_Mo group, which had 7220 up-regulated genes and 7993 down-regulated genes; the second was the Mo_vs_Cr group, which had 5313 up-regulated genes and 4160 down-regulated genes. Meanwhile, the group with the fewest DEGs was the CP_vs_Cr group, which only had 1829 up-regulated genes and 788 down-regulated genes.

### 3.3. GO Enrichment Analysis of DEGs

**Figure 3:**
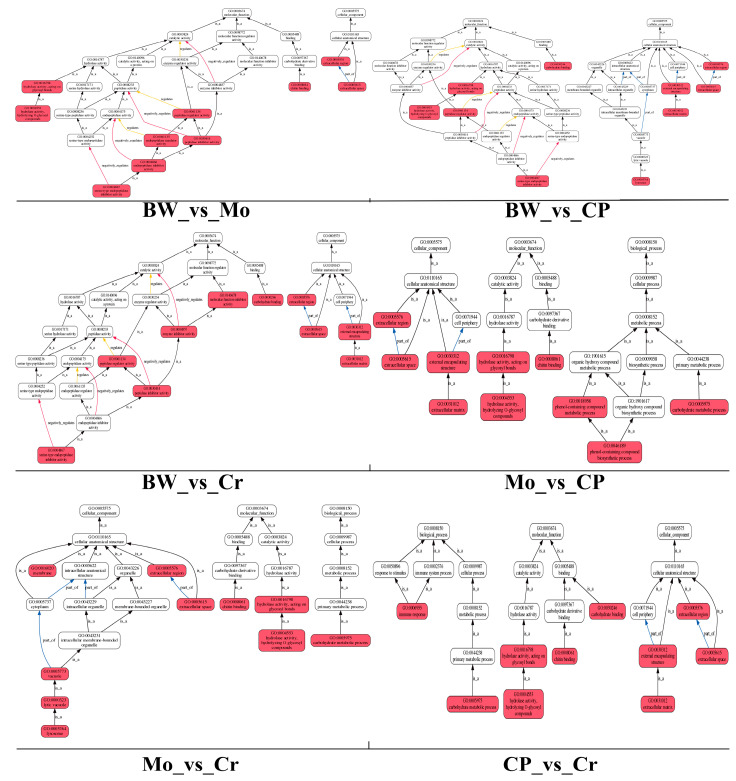
Th results of the GO functional enrichment-oriented analysis. Six comparison groups were analyzed, including BW_vs_Mo, BW_vs_CP, BW_vs_Cr, Mo_vs_CP, Mo_vs_Cr, and CP_vs_Crs.

Six comparison groups underwent GO (Gene Ontology) enrichment analysis to compare and evaluate the enrichment of metabolic pathways for differentially expressed genes. To clarify the enrichment pathways of differentially expressed genes in each comparison group, we conducted GO functional enrichment-oriented analysis. The results are shown in Figure 3. The DEGs from the six comparison groups were mainly rich in multiple pathways related to enzyme activity regulation (including peptidase, endopeptidase, and hydrolase), chitin metabolism, lysosomal functions, and so on. For example, in the comparison group (BW_vs_Mo group), which has the most DEGs, enrichment was mainly focused in chitin binding (GO: 0008061), peptidase regulator and inhibitor activity (GO: 0061134; GO: 0030414), and endopeptidase regulator activity (GO: 0061135).

### 3.4. KEGG Pathway Enrichment Analysis of DEGs

**Figure 4:**
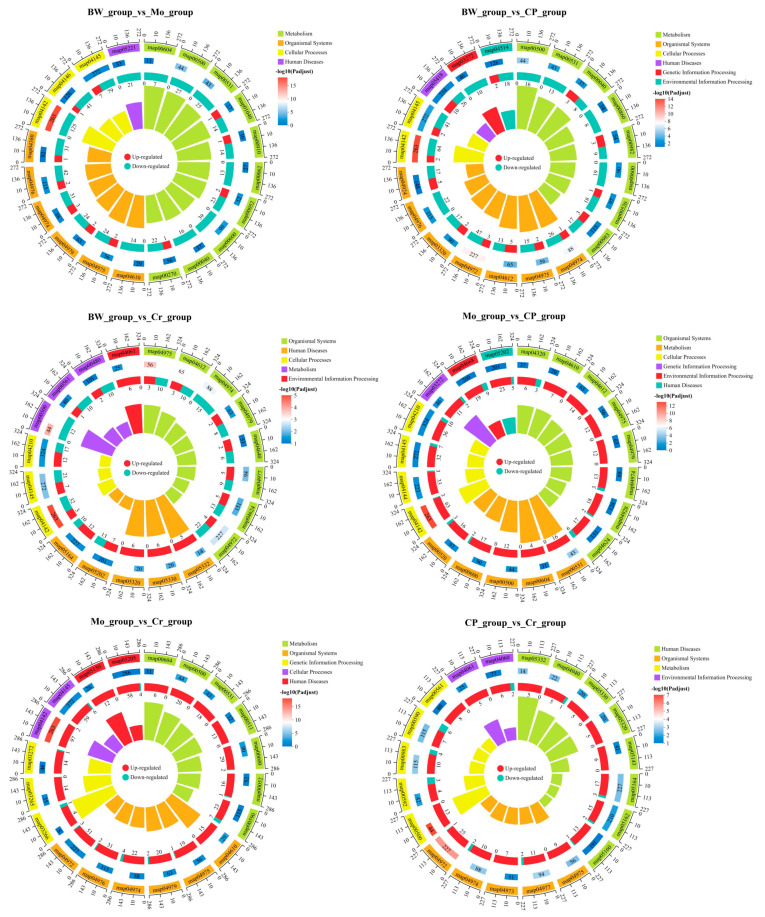
Multi-dimensional enrichment cycle diagram of KEGG pathway enrichment analysis. Six comparison groups are shown as follows: BW_vs_Mo, BW_vs_CP, BW_vs_Cr, Mo_vs_CP, Mo_vs_Cr, and CP_vs_Cr. “Map + numbers” represent KEGG pathway IDs. The enrichment diagram consists of four concentric circles: (1) The first circle: enriched pathway categories, with the outer circle representing the coordinate scale of the number of genes. Different colors represent different categories. (2) The second circle: the number of genes in this category denotes the background genes and the corresponding Q values or *p* values, where longer bars indicate more genes and redder colors indicate smaller p value. (3) The third circle: bar chart showing the proportions of up-regulated and down-regulated genes; the bottom shows the specific values. (4) The fourth circle: RichFactor values for each category (the number of differentially expressed genes in this category divided by the number of background genes).

The results of KEGG pathway enrichment analysis are shown in Figure 4. DEGs from the six comparison groups were concentrated in multiple similar signaling pathways, including map04145 (phagosome), map04142 (lysosome), map00500 (starch and sucrose metabolism), map04974 (protein digestion and absorption), and map04975 (fat digestion and absorption). The lysosome pathway (map04142) experienced the most significant enrichment among all groups except the CP_group_vs_Cr_group. Furthermore, the starch and sucrose metabolism pathway (map00500) was the most significantly enriched pathway in the CP_group_vs_Cr_group. In terms of classifying the enriched pathways, the BW_group_vs_CP_group and Mo_group_vs_CP_group had the highest number of enriched pathway categories (six), while the BW_group_vs_Mo_group and CP_group_vs_Cr_group had the fewest (four). However, when comparing the KEGG pathways enriched with all DEGs, although the enriched pathways were similar, the differences in the number of DEGs among each group were still very obvious.

### 3.5. qRT-PCR Results

**Figure 5:**
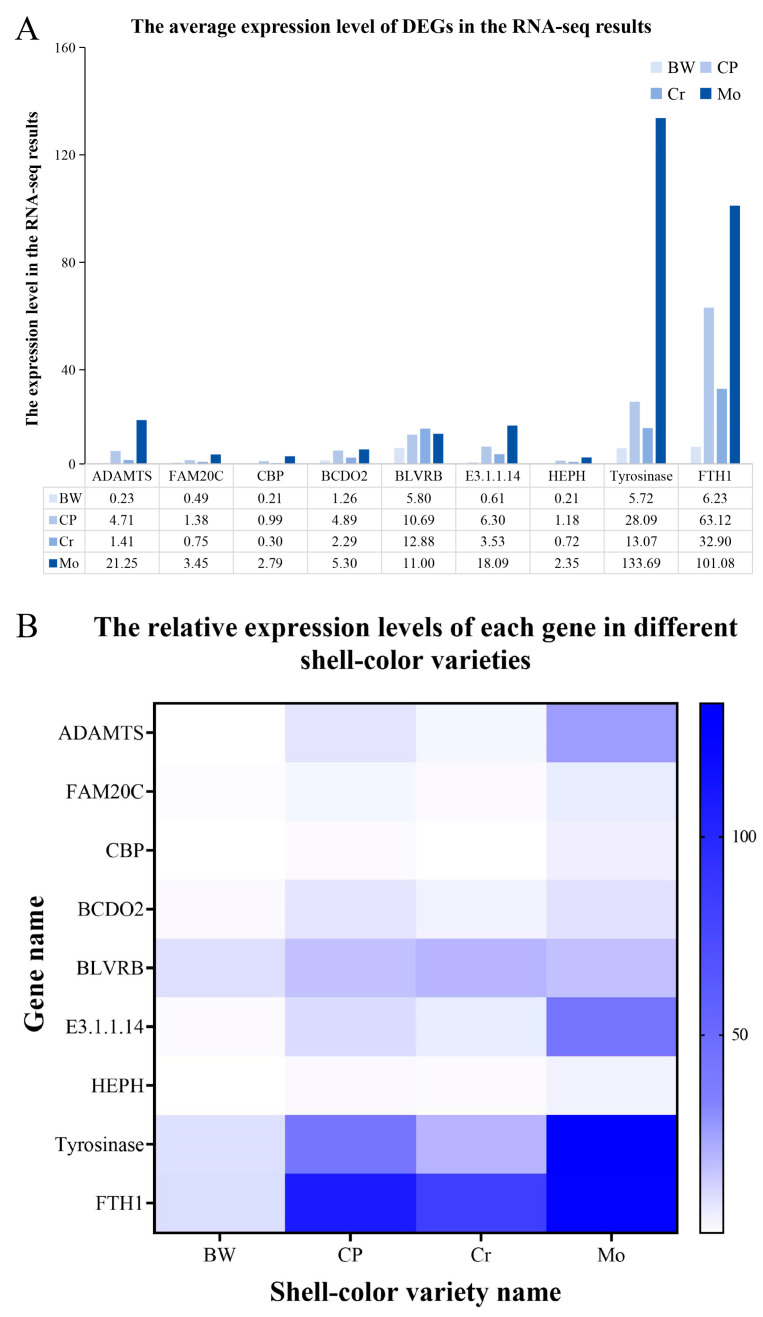
Comparison of RNA-seq and qRT-PCR detection results for nine DEGs. (**A**) Average expression levels of DEGs from RNA-seq results. The horizontal axis represents the names of these nine DEGs, and the vertical axis represents the expression levels of DEGs in the RNA-seq results. The numbers in the table represent the average expression level of each DEG in the four of shell color varieties (BW, CP, Cr, and Mo) (these DEG expression data were obtained from the results of this RNA-seq); (**B**) Heat map of expression levels of nine DEGs involved in different shell color varieties. The horizontal axis represents the names of the four types of shell color varieties (BW, CP, Cr, and Mo), and the vertical axis represents gene names. The color of the squares represents the relative expression level of each gene in the four shell color varieties; darker colors in the heat map indicate higher relative expression of the tested genes, and vice versa.

We selected nine DEGs (ADAMTS, FAM20C, CBP, BCDO2, BLVRB, E3.1.1.14, HEPH, tyrosinase, and FTH1) related to pigment metabolism for qRT-PCR detection. Primer information is listed in Table S3. The qRT-PCR analysis results were consistent with the RNA-seq results (Figure 5), suggesting that the transcriptome data generated by RNA-seq were reliable.

## 4. Discussion

In this study, RNA-seq analysis was conducted on four phenotypes of *S. cumingii* with different shell colors (Figure 1). According to earlier research reports, the metabolic levels of melanin and carotenoids significantly affect differences in shell and pearl colors. For example, the metabolic deposition of melanin significantly affects black and white colors of oyster shells (*Crassostrea gigas*) [11], and differences in melanin and carotenoid content significantly affect shell colors (gold and brown) in the noble scallop (*Chlamys nobilis*) [12]. Furthermore, porphyrin is another type of pigment that also plays a crucial role in determining shell color. Porphyrins are natural pigments that exist widely in the metabolic processes of animals and plants and can form chelates with various metal ions, producing various color phenotypes [24]. Studies have shown that porphyrin can directly affect shell color in Manila clams (*Ruditapes philippinarum*) [24]. Therefore, in our analysis, we also focused on these key points. After conducting a comparative analysis of multiple databases, we identified many DEGs involved in the melanin, carotenoid, and porphyrin metabolic pathways (Table 2), including ADAMTS, multiple tyrosinase genes, BCDO2, and FTH1. These DEGs exhibited the greatest differences between the BW and MO groups, with the majority showing significantly higher expression in the MO group; this has never been discovered in previous studies related to the shell color of *S. cumingii*.

The color differences in the shells of bivalve animals are likely due to calcium-based shells being more capable of integrating and combining with various organic pigment substances, thereby generating multiple different “combination patterns” and ultimately resulting in multiple color phenotypes [25]. This implies that the genetic characteristics of pigment metabolism-related genes in shellfish exhibit both conservation and diversity. Therefore, when studying the process of shell color formation, one can refer to the regulatory framework of pigment metabolism in other species. During the growth and development of melanocytes in fish, important regulatory pathways include SCF/KIT, WNT, MAPK, and cAMP [26]. Furthermore, some researchers have used in vitro l-tyrosine incubation assays of oyster mantle cells to verify that cAMP can mediate the melanin production pathway [11]. In our analysis, in the melanogenesis pathway (map04916), the expression levels of many TYR genes had significant differences among all groups. In addition, there were significant differences in the expression levels of key regulatory genes in the WNT, MAPK, and cAMP signaling pathways, such as WNT11, CALM, GNAI, FZD5_8, FZD9_10, GSK3B, and MAP2K1. This situation also occurs in the porphyrin metabolism pathway (map00860), in which heme is derived from the combination of porphyrins with divalent iron ions (Fe^2+^) and is hypothesized to affect shell color in Manila clams (*Ruditapes philippinarum*) [24]. Moreover, porphyrin is the most common precursor for the synthesis of heme and chlorophyll. Some studies have shown that porphyrin and chlorophyll metabolism, combined with calcium signaling pathway, are probably involved in shell color formation in Manila clams [27]. Therefore, both heme and chlorophyll may influence changes in shell color. E3.1.1.14, known as chlorophyllase, participates in the porphyrin metabolism pathway and regulates the metabolic degradation process of chlorophyll [28]. Meanwhile, during heme synthesis and metabolism, BLVRB acts as a regulatory gene that controls the conversion of biliverdin into bilirubin, thereby affecting heme content and ultimately influencing the accumulation of heme in the shell and associated color changes [29]. HEPH and FTH1 are regulatory genes that control the conversion of divalent iron ions (Fe^2+^) into trivalent iron ions (Fe^3+^). They also transport iron elements, the content of which can directly affect the formation of heme [24,30]. These genes all showed significant differential expression in this study. Furthermore, BCDO2 is a key enzyme in the carotenoid metabolism pathway (map00906) [31], and its accumulation in the mantle of the red shell hard clam (*Meretrix meretrix*) can significantly affect color changes in their shells [32]. In our study, BCDO2 also had significant differential expression across all six comparison groups.

Melanin, carotenoids, and porphyrins can produce various colors by combining with certain metal ions, such as iron, which plays a very important role in synthesizing heme and melanin. The combination of porphyrin with Fe^2+^ produces heme [24]. Meanwhile, heme is the substrate for heme synthase and, as an iron carrier, directly affects the synthesis process of melanin in melanosomes by regulating catalase, peroxidasin, and TYR [24]. Therefore, the absorption and metabolism of trace minerals can also significantly affect the color of shells and pearls [33]. In the mineral absorption pathway (map04978), we also found many DEGs, such as TRPV6 (which promotes metabolic absorption of calcium ions) [34], HCP1 (also called SLC46A1, which is involved in the metabolic absorption of iron ions) [35], FTH1 (which is a core regulatory gene that regulates metabolism and absorption of iron ions) [30], HEPH (a core regulatory gene that regulates the metabolism and absorption of iron ions) [30], Zip4 (also called SLC39A4, which is involved in the absorption and metabolism of zinc ions) [36], and ATP1A and ATP1B, which are involved in regulating the metabolic exchange of sodium and potassium ions [37]. Interestingly, these genes all showed significant down-regulation in the BW_vs_Mo group, which might potentially explain the difference in shell and pearl color between them (blue-white and modena) (Figure 1).

These findings may explain shell color variation in *S. cumingii*, which may be due to significant differential expression of genes related to pigment metabolism and mineral absorption. Moreover, our results suggest that melanin, carotenoids, porphyrins, and heme likely played important roles in shell color formation, which is consistent with previous reports.

## 5. Conclusions

This study conducted a transcriptome analysis of four distinct color morphs of *Sinohyriopsis cumingii*. By studying expression differences in DEGs related to pigment metabolism and mineral absorption, we were able to explore potential reasons for shell color variation in *S. cumingii*. Our results are consistent with previous reports and are supported by expression patterns. This study also reveals the significant roles of melanin, carotenoids, porphyrins, and heme in shell color formation, which might be the underlying reason why *S. cumingii* produces pearls of different colors. On the other hand, the coloration process of shells and pearls is primarily regulated by mantle activity, and conducting sequencing analysis solely on total RNA to study the color-changing process of shells and pearls may introduce a higher level of complexity. Thus, the causal relationship between the identified DEGs associated with pigment metabolism and mineral absorption and the actual coloration of shells and pearls requires further investigation and validation. In future studies, we will integrate examinations of mantle secretions with targeted research on pigment metabolism-related genes to more accurately elucidate the mechanisms underlying shell and pearl color formation.

## Figures and Tables

**Table 1:** Sequencing data statistics for 12 RNA libraries of *Sinohyriopsis cumingii*.

Sample	Raw Reads	Raw Bases	Clean Reads	Clean Bases	Error Rate (%)	Q30 (%)	GC Content (%)	Mapped Ratio (%)
BW_1	41,164,840	6,215,890,840	40,913,660	6,139,659,156	0.0114	96.97	39.35	87.74
BW_2	41,970,544	6,337,552,144	41,715,984	6,265,297,571	0.0115	96.79	39.89	88.64
BW_3	39,516,106	5,966,932,006	39,278,874	5,897,143,366	0.0115	96.81	39.92	88.85
Mo_1	39,199,452	5,919,117,252	38,987,226	5,860,304,931	0.0115	96.78	42.47	88.88
Mo_2	40,781,074	6,157,942,174	40,518,708	6,084,425,927	0.0115	96.78	43.03	89.67
Mo_3	38,472,030	5,809,276,530	38,261,548	5,746,516,575	0.0116	96.69	42.48	88.78
CP_1	39,582,318	5,976,930,018	39,340,546	5,905,289,296	0.0114	96.93	40.4	88.17
CP_2	45,697,584	6,900,335,184	45,426,634	6,827,017,417	0.0114	96.95	40.7	88.28
CP_3	39,820,252	6,012,858,052	39,580,722	5,943,966,892	0.0115	96.82	40.92	88.15
Cr_1	40,254,568	6,078,439,768	40,015,880	6,008,432,752	0.0115	96.86	39.8	88.27
Cr_2	41,604,442	6,282,270,742	41,371,482	6,214,076,897	0.0114	96.95	40.11	88.57
Cr_3	44,078,990	6,655,927,490	43,814,922	6,581,823,448	0.0115	96.85	39.82	87.86

**Table 2:** DEGs that are significantly associated with shell color difference in *Sinohyriopsis cumingii*.

Latent Function	Sequence ID	Gene Name
Melanin-related	TRINITY_DN10154_c0_g1	ADAMTS
	TRINITY_DN11197_c0_g1	FAM20C
	TRINITY_DN17113_c0_g1	Tyrosinase
	TRINITY_DN9065_c0_g2	CBP
	TRINITY_DN955_c0_g2	Tyrosinase-like protein
Carotenoid-related	TRINITY_DN10792_c0_g1	BCDO2
Porphyrin-related	TRINITY_DN12413_c0_g1	FTH1
	TRINITY_DN14348_c0_g2	BLVRB
	TRINITY_DN20174_c0_g1	FTH1
	TRINITY_DN29673_c0_g1	E3.1.1.14
	TRINITY_DN61394_c0_g1	HEPH
Mineral-related	TRINITY_DN10483_c0_g1	SLC31A1
	TRINITY_DN13469_c0_g1	SLC46A1
	TRINITY_DN13884_c0_g1	SLC9A3
	TRINITY_DN14110_c0_g1	ATP1A
	TRINITY_DN16830_c1_g2	NR1F1
	TRINITY_DN20174_c0_g1	FTH1
	TRINITY_DN21109_c0_g2	Zip4/SLC39A4
	TRINITY_DN23691_c0_g1	Plasma membrane calcium ATPase
	TRINITY_DN24296_c0_g2	SLC26A6
	TRINITY_DN3032_c1_g1	SLC34A
	TRINITY_DN34788_c0_g1	ATP1B
	TRINITY_DN36270_c0_g1	TRPV6
	TRINITY_DN40313_c0_g1	NR1F1
	TRINITY_DN50867_c0_g1	SLC39A12/ZIP12
	TRINITY_DN61394_c0_g1	Hephaestin-like
	TRINITY_DN61394_c0_g1	HEPH
	TRINITY_DN8266_c0_g1	VDR/NR1I1

## Data Availability

The raw transcriptome data have been uploaded to the NCBI (project number: PRJNA1359545); the NCBI accession numbers are SRR35992403; SRR35992404; SRR35992405; and SRR35992406. Other data that support the findings of this study are available from the corresponding author upon reasonable request.

## Supplementary Materials

The following supporting information can be downloaded at https://www.mdpi.com/article/10.3390/ani16030416/s1, Table S1: Expression difference statistics of DEGs among the six comparison groups. Table S2: Annotation information for all DEGs. Table S3: QRT-PCR primer sequence information.
