# Supplementary material for: Transcriptome Analysis of Differentially Expressed Genes in Freshwater Pearl Mussel (Sinohyriopsis cumingii) with Four Different Shell Colors
Source: Animals (Basel). 2026 Jan 29;16(3):416. doi: 10.3390/ani16030416 (PMC12897141; doi:10.3390/ani16030416)
Supplement: Supplementary file 1 [file animals-16-00416-s001.zip › Supplementary material legend.pdf]

Supplementary material information:

Table S1. Expression difference statistics of DEGs in the six comparison groups.

Table S2. All annotation information for the DEGs.

Table S3. QRT-PCR primer sequence information.
